# Protocol to improve quality of life, functionality, and exercise capacity in patients with axillary web syndrome after breast cancer: a randomized clinical trial

**DOI:** 10.1007/s00520-025-09815-w

**Published:** 2025-08-14

**Authors:** Jesús Baltasar González-Rubino, Rocío Martín-Valero, María Jesús Vinolo-Gil

**Affiliations:** 1 Department of Physiotherapy, Faculty of Health Sciences, CTS-1071 Research Group, University of Malaga, Malaga, Spain; 2 Rehabilitation Clinical Management Unit, Hospital Punta Europa, Campo de, Oeste Health District, 11202 Algeciras, Cadiz, Spain; 3 Department of Nursing and Physiotherapy, University of Cadiz, 11009 Cadiz, Spain; 4 Rehabilitation Clinical Management Unit, Interlevels-Intercenters Hospital Puerta del Mar, Hospital Puerto Real, Cadiz Bay-La Janda Health District, 11006 Cadiz, Spain; 5 Department Biomedical Research and Innovation Institute of Cadiz (INiBICA), Research Unit, Puerta del Mar University Hospital, University of Cadiz, 11009 Cadiz, Spain

**Keywords:** Axillary web syndrome, Breast cancer, Physiotherapy, Stretching, Lymphatic system, Physical therapy

## Abstract

**Objectives:**

To improve functionality, quality of life, and exercise capacity in women who underwent breast cancer surgery and suffered axillary web syndrome (AWS) as a sequela.

**Design:**

Prospective, randomized, single-blind clinical trial.

Setting.

Single-center study.

Participants.

Forty-six post-breast cancer surgery patients with AWS had restricted functionality, quality of life, and exercise capacity.

Interventions.

Stretching combined with manual therapy and scar massage to release adhesion and lymphatic cord during 15 physiotherapy sessions of 30–40 min each.

Main outcome measures.

Functionality was evaluated with DASH and Constant Scale, exercise capacity with IPAQ, and quality of life with EORTC QLQ-BR23.

Participants were allocated to either intervention or control group using the Excel randomization tool. Blinding of the therapist was not possible due to the study’s nature.

**Results:**

Twenty-five patients were assigned to the intervention group and twenty-three to the control group, becoming twenty-four and twenty-two, respectively, for analysis.

Significant differences were detected in functionality and quality of life. The intervention effect varied over time with a 95% confidence interval (alpha 0.05) and a statistical power of 90% (beta 0.1). Comparisons between groups showed significant differences in favor of the intervention group at 30, 60, and 90 days of follow-up.

**Conclusion:**

Stretching combined with scar massage and manipulative tissue release techniques improved functionality and quality of life in AWS patients. This physiotherapy technique could be considered the treatment of choice for this surgical sequela.

Trial registration.

ClinicalTrials.gov Registry (NCT05115799) on June 10, 2021, and the approval of the Andalucía Ethics Committee (PEIBA code 1909-N1-21, reg. number 171.21).

**Supplementary Information:**

The online version contains supplementary material available at 10.1007/s00520-025-09815-w.

## Introduction

Breast cancer is one of the most prevalent types of cancer worldwide, particularly among women. In 2020, 2 million new cases were diagnosed globally [1]. In the USA, it ranks as the second most common cancer after skin cancer, accounting for about 30% of all new female cancer cases each year [2].

Axillary web syndrome (AWS) is one of the most unknown negative sequelae which does affect many breast cancer patients who underwent surgery [3] (up to 85.4% according to Yeung et al. [4]). It can also be identified with the following terms: “cording,” “axillary string,” “vascular string,” “lymphatic cord,” “fibrous banding,” or “Mondor’s disease” [5].

There are some risk factors related to the development of AWS: type of surgery (complexity or extensiveness of surgery, type of surgical procedure, and number of gland nodes removed) [3–8], a lower BMI, younger age, and healing complications (numbness after intercostobrachial nerve injury or haematoma) [4]. There is no known link between AWS and lymphoedema, being these two considered independent from each other [4, 5, 9].

AWS symptoms are varied, ranging from pain, reduction of the shoulder range of movement to functionality, and quality of life decrease [4, 7, 10, 11]. If it is not treated effectively, it may cause adhesive capsulitis and myofascial pain syndrome [12]. The axillary cords are always found in the axilla, and some may extend along the arm down to the elbow. Normally, down to the antecubital fossa and into the forearm. In some rare cases, they may extend all the way down to the radial aspect of the wrist and into the base of the thumb [11].

First signs and symptoms can be observed from the eighth to the twelfth week after surgery. Usually, it disappears after 3–4 months after surgery. Nevertheless, there are situations where AWS is present for up to 24 months after surgery [13, 14]. Therefore, delaying the radiotherapy treatment [10, 11]. Very occasionally, there are reported cases of late recurrence [7].

Palpation, visual inspection, and reported symptoms are some of the procedures to diagnose AWS. The thickness of the adipose tissue may pose a challenge in palpation of the cord [4]. Baggi et al. created in 2012 a self-assessment questionnaire, which was added to physical evaluation for the diagnosis [12]. Although lymphoscintigraphy can be considered gold standard for lymphatic accurate diagnosis [15], nuclear magnetic resonance and ultrasound are also valid methods to confirm the presence of the cord [16]. Thickness of AWS and its disorganization can be assessed through ultrasound according to some studies [17].

No treatment of choice for AWS has yet been described [18]. Physical therapy has proven its benefits when applied early after surgery to prevent surgical sequelae without complications [8, 17, 19]. Moreover, it helps to improve AWS symptoms. There is a wide variety of scientific publications related to physical therapy [3]: articles describing exercises combined with manual lymphatic drainage [6, 7, 18], stretching and snapping maneuvers as a treatment strategy [14], other studies use strength and endurance exercises [20], or even the application of myofascial release [16, 17]. Despite this, the conclusion is according to systematic reviews and narrative reviews, that there is a need for more quality randomized clinical trials to define a treatment of choice for AWS [5, 14, 21].

## Objective

To improve functionality, quality of life, and exercise capacity in women who have undergone breast cancer surgery and suffer AWS.

### Hypothese

With the application of this physiotherapy treatment, functionality, quality of life, and exercise capacity are sought to be improved significantly in patients suffering from AWS in comparison to current strategies.

## Methods

This is a two-arm randomized clinical trial. This research uses the guidelines on Standards for Quality Improvement and Excellence in Reporting and Consolidated Standards of Reporting Trials (CONSORT) [22].

**Fig. 1:**
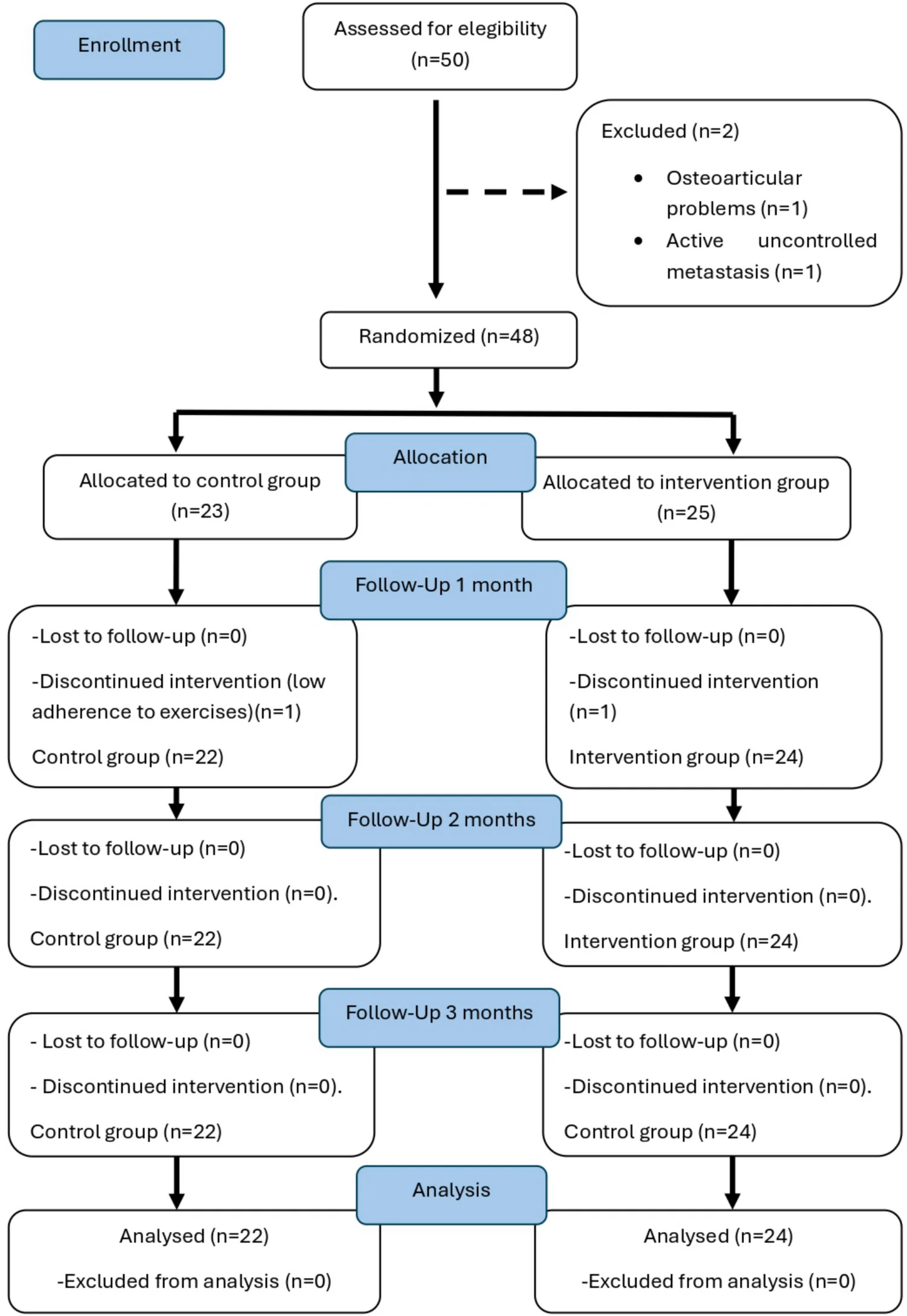
CONSORT flow chart

The Standard Protocol Items: Recommendations for Interventional Trials checklist is provided in Fig. 1. The research procedure was approved by the Andalucía Ethics Committee on Human Research (PEIBA code 1909-N1-21, reg. number 171.21) and with Clinical Trial Registration number: ClinicalTrials.gov Registry (NCT05115799). The study was conducted in accordance with the amended Declaration of Helsinki.

*Informed consent statement: an informed consent form was prepared and signed by all the subjects participating in the study. Prior to signing, they were provided with sufficient information about the objectives and procedures of the research. They were also informed that they could revoke their consent at any time, without the need to justify their decision and without any prejudice. All necessary permits were obtained from the relevant institutions for the development of the study.

*Patient and public involvement: Patients or the public were not involved in the design, conduct, or reporting. However, once the study is published, the results will be shared directly with the patients. They will be informed through a dedicated website (@unidadlinfedemaalgeciras2267) and will receive a summary of the findings in a study newsletter, written in a way that is accessible to a non-specialist audience. This ensures that participants are fully informed about the outcomes and the impact of the research.

*Participants:

- Inclusion criteria: Women over 18 years of age, who underwent breast cancer surgery and AWS, appeared within 1 year after such surgery. Patients who stated that AWS was palpable, painful, and with reduction on functionality and detriment in their quality of life. All participants are patients within Campo de Gibraltar West Health Management Area.
- Exclusion criteria: (patients with one or more of the following criteria) Patients with psychological or neurological disorders, with active metastases without chemotherapy treatment, rheumatological or osteoarticular problems limiting joint mobility, and to receive a different physiotherapy treatment for AWS than the one prescribed in the study.
- Withdrawal criteria: A mild adverse event; a serious, unexpected, or clinically relevant adverse event or not achieving the suggested exercise performance at least six days a week.

Interventions

### Control group

Participants are trained by the physiotherapist in the first consultation on Codman’s pendulum exercises and on self-assisted stretching in seated and standing positions. The stretches are explained to the patient by the specialist physiotherapist and initially performed together to correct possible errors. Advice is given on possible postural compensations. The importance of performing these exercises daily is explained. At least 15 repetitions of each exercise holding the stretch for 15–20 s. The exercises are described in Appendix 1 [23].

After diagnosis, patients attend group therapy twice where exercises are repeated and corrected. The groups are limited to less than 6 patients to ensure quality care. In addition, an informative talk about lymphoedema and AWS is delivered, in which preventive measures (usual care) are specified. Follow-up is carried out monthly for the next 3 months. At each review, adherence to the program is assessed, confirming that the participant performs the exercises effectively at least 6 days a week. In the case any patient in the control group received additional treatment for AWS or any physical therapy treatment, this must be notified to the specialist physiotherapist who may consider the exclusion from the study. Patients were requested not to initiate any further exercise training that was not performed prior their participation in this study.

### Intervention group

Participants receive the same training as control group patients, but intervention group patients receive 15 physiotherapy sessions within three weeks, that is 5 sessions per week delivered by the same specialist physiotherapist. Each session lasts 30–40 min. During the sessions, the patient is placed on the couch in the supine position. The physiotherapist applies stretches to the affected arm, considering patient’s tolerance, the pain never beyond level 6 in the VAS scale. Initial warm-up is performed with self-assisted mobility of the upper limb and bringing the arm to the maximum flexion or abduction that the patient can tolerate without the appearance of elevated pain. The elbow is usually in almost full extension (if the cord allows such a position), supination, wrist extension, and thumb opposition. For no more than 30 s in this position, the physiotherapist palpates the cord and works through it, with friction similar to the scar massage maneuver at the level of the axillary or mammary scar, where the AWS usually originates. This manipulative technique of friction is also applied to the cord in the area which the patient describes as discomforting. The friction is carried out perpendicular and longitudinally to the cord. Longitudinal manipulations are more frequent than perpendicular, and they are based on pulling the skin distally and in the areas where palpation of the cord is most uncomfortable for the patient. The maneuver should be gentle to avoid the appearance of skin erythema. Out of the 30–40 min for each session, the first 5–10 min are dedicated to warm-up performing pendular exercise. As soon as warm up is completed, the patient is placed in the supine position and the exercises described in Appendix 2 are performed [23]. For the next 25–30 min, the patient assisted by the specialist physiotherapist will perform stretches described in Appendix 2 in 30 s slots. Meanwhile, the specialist physiotherapist palpates and performs the aforementioned described maneuvers. In the case any patient in the intervention group received additional treatment for AWS or any physical therapy treatment, this must be notified to the specialist physiotherapist who may consider the exclusion from the study. Patients were requested not to initiate any further exercise training that was not performed prior their participation in this study.

### Outcomes

As a first step, sociodemographic data is gathered: such as age, marital status, employment status, or educational level. Also, whether she has ever become a mother and when. Regarding lifestyle, whether she practices sport and how often. Whether she lives in an urban or rural area. It is also considered whether she smokes or not. Regarding medical records, body mass index (BMI), type of tumor, when the axillary web syndrome appeared, number of lymph nodes removed, whether her surgery was radical or conservative, whether she has received radiotherapy, and finally whether the patient received breast reconstruction or not. The assessment for all the outcomes was carried out by the same specialist physiotherapist.

Axillary cord syndrome: The presence of lymphatic cord was assessed by observation and palpation by the assessor. Physical exam performed as suggested in previous research: Patient laying in supine position with elbow extended and the shoulder in maximum abduction. The assessor observes and palpates the beads, including the armpit, down the upper arm from the armpit to the antecubital space, and through the forearm to the base of the thumb [4].

Constant Scale: According to the Spanish Society for Shoulder and Elbow Surgery (SECHC), the Constant Scale assesses pain, functionality for daily life activities, joint mobility, and shoulder strength. Also, it considers the laterality and the time it takes for the patient. The score ranges from 0 to 100 points, being 100 the optimal condition for the shoulder [24].

Quick-Dash (DASH): The Disabilities of the Hand, Arm and Shoulder (DASH) questionnaire is a specific instrument for measuring the quality of life related to health problems in the upper limbs. It is validated in Spanish, and it consists of 30 questions. The final score calculation is relatively complicated. In order to calculate the scores, it is necessary to have answered at least 27 out of the 30 questions. The final score is obtained by calculating the arithmetic means of the questions answered minus 1 times 25. The DASH questionnaire has excellent reproducibility and high sensitivity, being able to detect small changes. The scale ranges from 30 to 150 points. Thirty points means good shoulder functionality and 150 non-functional shoulder.

It has two optional subsections where sports and work functionality can be assessed [25].

International Scale of Physical Activity (IPAQ): The main use of the IPAQ (International Physical Activity Questionnaire) worldwide is aimed at monitoring and investigation purposes. It is an instrument designed mainly for the “monitoring” of physical activity performed by the adult population and their perception of their health. Its aim is to learn about the kind of physical activity that people perform as part of their daily activities. The questions are focused on the time the patient spends being physically active during the previous 7 days. The patient should consider the activities he/she does as part of work, gardening, at home, leisure, and moving from one place to another during their rest, exercise, or sport [26].

EORTC-QLQ-BR23 questionnaire: It consists of a validated questionnaire consisting of 30 questions. The first 28 questions are scored from 1 to 4, with the highest values being those that show greater difficulty when carrying out the activity for which they are asked or the worst state of health. One hundred twenty-six points is the highest score and means excellent health. Zero points means the worst health [27].

Finally, there are two general questions about the state of health and quality of life that score from 1 to 7, the highest value being the best state of health and quality of life. The vast majority of questions refer to the previous week.

Sample size: To determine the sample size for comparing two means, we aimed to detect a clinically meaningful difference in healing time for AWS, measured in days, between intervention and control group. Both current literature and expert input agreed that a difference of at least 15 days would be clinically relevant. Given that healing time is a quantitative outcome, we chose a comparison of means between the two groups as the basis for the sample size calculation.

For this study, we set a confidence level of 95% (alpha = 0.05) and a statistical power of 90% (beta = 0.1) while accounting for an anticipated loss to follow-up of 20%. Based on a standard deviation of 14 days in healing time for standard treatments, we used the pwr package in R to calculate the necessary sample size. To detect a difference of 15 days or more between groups with the specified confidence and power, 18 subjects per group were required. Adjusting for the potential 20% follow-up loss, the total sample size needed was determined to be 46 participants.

- Randomization allocation: Before commencing the study, Excel program “randomization” tool was used to provide an allocation sequence list for patients from 1 to 46. The position on the list would be assigned on arrival order. As patients arrived at the clinic for the first evaluation, they were informed about the study. If they agreed to participate, and after giving written consent, their physiotherapist would enroll the patient to either control or intervention group, according to the previously mentioned Excel list.
- Masking: Blinding could not be performed due to the nature of the intervention because participants can clearly see whether they are being intervened at the health center by the specialist physiotherapist or not, as the control group patients solely perform the activities required by the specialist physiotherapist at home without in-person physiotherapy treatment. Physiotherapy studies often face challenges in masking both the intervention and the therapist.
- Statistical analysis: Descriptive statistics were computed as means and standard deviations for continuous outcomes, and medians with interquartile ranges for non-normally distributed data. Proportions and 95% confidence intervals were calculated for categorical outcomes. Cohort homogeneity at baseline was assessed by comparing all measured outcomes between patient groups using nonparametric Wilcoxon tests. *p*-values were adjusted using the FDR method to control for type I error (Table 1).

**Table 1:** Descriptive analyses of the variables considered in the study and multiple comparisons based on patient groups. The mean and standard deviation (X ± SD) are presented for quantitative variables and percentage with 95% confidence interval [% (95% CI)] for qualitative variables. *p*-values have been adjusted using the FDR method

	**Control**	**Intervention**	***p*-value**
Age			0.984
	*N* = 22	*N* = 24	
	49.09 ± 8.7	50.54 ± 10.61	
Marital status			0.688
Single	*N* = 2	*N* = 7	
	9.09% (2.53–27.81%)	29.17% (14.91–49.17%)	
Married/domestic partnership	*N* = 16	*N* = 12	
	72.73% (51.85–86.85%)	50% (31.43–68.57%)	
Divorced/separated	*N* = 3	*N* = 4	
	13.64% (4.75–33.33%)	16.67% (6.68–35.85%)	
Widowed	N = 1	N = 1	
	4.55% (0.81–21.8%)	4.17% (0.74–20.24%)	
Education level			0.447
No education	*N* = 1	*N* = 0	
	4.55% (0.81–21.8%)	0% (1.39e-15–13.8%)	
Primary	*N* = 3	*N* = 5	
	13.64% (4.75–33.33%)	20.83% (9.24–40.47%)	
Secondary	*N* = 14	*N* = 8	
	63.64% (42.95–80.27%)	33.33% (17.97–53.29%)	
University	*N* = 4	*N* = 11	
	18.18% (7.31–38.52%)	45.83% (27.89–64.93%)	
Residence			1
Rural area	*N* = 0	*N* = 1	
	0% (1.39e-15–14.87%)	4.17% (0.74–20.24%)	
Urban area	*N* = 22	*N* = 23	
	100% (85.13–100%)	95.83% (79.76–99.26%)	
Children			0.518
Yes	*N* = 20	*N* = 17	
	90.91% (72.19–97.47%)	70.83% (50.83–85.09%)	
No	*N* = 2	*N* = 7	
	9.09% (2.53–27.81%)	29.17% (14.91–49.17%)	
Mother over 30 years			0.632
Yes	*N* = 6	*N* = 11	
	27.27% (13.15–48.15%)	45.83% (27.89–64.93%)	
No	*N* = 16	*N* = 13	
	72.73% (51.85–86.85%)	54.17% (35.07–72.11%)	
Smoker			0.594
Yes	*N* = 2	*N* = 7	
	9.09% (2.53–27.81%)	29.17% (14.91–49.17%)	
No	*N* = 15	*N* = 14	
	68.18% (47.32–83.64%)	58.33% (38.83–75.53%)	
Ex-smoker	*N* = 5	*N* = 3	
	22.73% (10.12–43.44%)	12.5% (4.34–31%)	
Weight (kg)			0.547
	*N* = 22	*N* = 24	
	62.07 ± 10.97	58.87 ± 8.25	
Height (cm)			0.426
	*N* = 22	*N* = 24	
	1.59 ± 0.06	1.62 ± 0.06	
BMI			0.473
	*N* = 22	*N* = 24	
	24.63 ± 4.53	22.31 ± 2.82	
Employment status			0.714
Active	*N* = 12	*N* = 15	
	54.55% (34.66–73.08%)	62.5% (42.71–78.84%)	
Home care	*N* = 0	*N* = 0	
	0% (1.39e-15–14.87%)	0% (1.39e-15–13.8%)	
Unemployed	*N* = 3	*N* = 3	
	13.64% (4.75–33.33%)	12.5% (4.34–31%)	
Retired	*N* = 0	*N* = 3	
	0% (1.39e-15–14.87%)	12.5% (4.34–31%)	
Sporting activities			0.820
Yes	*N* = 13	*N* = 17	
	59.09% (38.73–76.74%)	70.83% (50.83–85.09%)	
No	*N* = 9	*N* = 7	
	40.91% (23.26–61.27%)	29.17% (14.91–49.17%)	
Type of cancer			1
Invasive	*N* = 19	*N* = 22	
	86.36% (66.67–95.25%)	91.67% (74.15–97.68%)	
Metastatic	*N* = 0	*N* = 0	
	0% (1.39e-15–14.87%)	0% (1.39e-15–13.8%)	
Non-invasive	*N* = 1	*N* = 1	
	4.55% (0.81–21.8%)	4.17% (0.74–20.24%)	
Nodes removed			0.874
	*N* = 22	*N* = 24	
	4.45 ± 4.43	4.79 ± 4.8	
Type of surgery			1
Conservative	*N* = 15	*N* = 17	
	68.18% (47.32–83.64%)	70.83% (50.83–85.09%)	
Radical	*N* = 7	*N* = 7	
	31.82% (16.36–52.68%)	29.17% (14.91–49.17%)	
Breast reconstruction			0.928
No	*N* = 18	*N* = 18	
	81.82% (61.48–92.69%)	75% (55.1–88%)	
Prosthesis	*N* = 4	*N* = 6	
	18.18% (7.31–38.52%)	25% (12–44.9%)	
Radiotherapy			0.859
Yes	*N* = 19	*N* = 20	
	86.36% (66.67–95.25%)	83.33% (64.15–93.32%)	
No	*N* = 3	*N* = 4	
	13.64% (4.75–33.33%)	16.67% (6.68–35.85%)	
Sessions radiotherapy			0.790
	*N* = 18	*N* = 18	
	13 ± 5.71	12.5 ± 4.93	
Chemotherapy			0.708
Yes	*N* = 16	*N* = 14	
	72.73% (51.85–86.85%)	58.33% (38.83–75.53%)	
No	*N* = 6	*N* = 10	
	27.27% (13.15–48.15%)	41.67% (24.47–61.17%)	
Cycles chemotherapy			0.828
	*N* = 16	*N* = 14	
	14.56 ± 3.76	14.79 ± 4.37	
Monoclonal antibodies			0.949
Yes	*N* = 6	*N* = 8	
	27.27% (13.15–48.15%)	33.33% (17.97–53.29%)	
No	*N* = 16	*N* = 16	
	72.73% (51.85–86.85%)	66.67% (46.71–82.03%)	
Hormone therapy			0.663
Yes	*N* = 15	*N* = 20	
	68.18% (47.32–83.64%)	83.33% (64.15–93.32%)	
No	*N* = 7	*N* = 4	
	31.82% (16.36–52.68%)	16.67% (6.68–35.85%)	
Cancer stage (TNM scale) T			0.965
T1	*N* = 12	*N* = 13	
	54.55% (34.66–73.08%)	54.17% (35.07–72.11%)	
T2	*N* = 8	*N* = 7	
	36.36% (19.73–57.05%)	29.17% (14.91–49.17%)	
T3	*N* = 1	*N* = 3	
	4.55% (0.81–21.8%)	12.5% (4.34–31%)	
T4	*N* = 1	*N* = 0	
	4.55% (0.81–21.8%)	0% (1.39e-15–13.8%)	
TX	*N* = 0	*N* = 1	
	0% (1.39e-15–14.87%)	4.17% (0.74–20.24%)	
Stage cancer (TNM scale) N			0.970
N0	*N* = 11	*N* = 12	
	50% (30.72–69.28%)	50% (31.43–68.57%)	
N1	*N* = 8	*N* = 11	
	36.36% (19.73–57.05%)	45.83% (27.89–64.93%)	
N2	*N* = 1	*N* = 1	
	4.55% (0.81–21.8%)	4.17% (0.74–20.24%)	
N3	*N* = 1	*N* = 0	
	4.55% (0.81–21.8%)	0% (1.39e-15–13.8%)	
**NX**	*N* = 1	*N* = 0	
	4.55% (0.81–21.8%)	0% (1.39e-15–13.8%)	
Stage cancer (TNM SCALE) M			0.965
M0	*N* = 10	*N* = 12	
	45.45% (26.92–65.34%)	50% (31.43–68.57%)	
MX	*N* = 12	*N* = 12	
	54.55% (34.66–73.08%)	50% (31.43–68.57%)	
Time since diagnosis (months)			0.615
	*N* = 22	*N* = 24	
	0.82 ± 1.84	1.58 ± 3.02	
Time from surgery to appearance of lymphatic thrombus (days)			0.965
	*N* = 22	*N* = 24	
	38.64 ± 58.38	60.92 ± 99.59	
Dominant hand			1
Right	*N* = 21	*N* = 22	
	95.45% (78.2–99.19%)	91.67% (74.15–97.68%)	
Left	*N* = 1	*N* = 2	
	4.55% (0.81–21.8%)	8.33% (2.32–25.85%)	
Number of exercises per week			0.708
	*N* = 22	*N* = 24	

For quantitative outcomes, Wilcoxon tests were conducted. *P-*value Probability value, *N *Population size ± Range of values or a confidence interval, * TNM *staging system is a method for classifing cancer base on the extent of the tumor, the presence of regional lymph node involvement, and the presence of distant metastasis *X *Mean, *SD *Standard Deviation.

To evaluate the effect of physiotherapeutic treatment on the quality of life of breast cancer surgery patients, mixed-effects regression models were employed. These models accounted for both fixed effects (e.g., intervention group, time, and covariates such as age, BMI, and tumor stage) and random effects (individual variability). This approach controlled for baseline differences and allowed for a more accurate estimation of treatment effects. Models were adjusted for several outcome measures, including the Constant-Murley, DASH, IPAQ, and EORTC QLQ-C30, and interactions between the intervention group and time were included. Model fitting followed Zuur and Ieno’s protocol [28], with random effects for patient identity and fixed effects selected via backward elimination. Model comparison was conducted using corrected Akaike Information Criterion (AICc), and the significance of categorical factors was assessed via ANOVA, with post hoc comparisons using the emmeans package. Assumptions were verified through residual analysis and Shapiro–Wilk (normality) and Levene (homoscedasticity) tests.

All analyses were performed using R v.4.4.2 [29]. Statistical significance was set at α = 0.05.

Assessment procedure (adherence monitoring)

If during regular post-surgery checkups AWS was diagnosed, the patient was recruited for the study after signing informed consent. Patients were requested to answer a clinical interview in person on day one, day thirty, sixty, and on day ninety as aforementioned in the outcomes section. Performance and adherence were assessed from day 30 onwards.

In-person appointment was reminded via telephone a few days beforehand. In case the patient could not attend, a three-day window was considered.

On appointment day, the specialist physiotherapist showed the patient how to perform the exercises and checked that the patient was able to perform them correctly.

Patients were required to perform those exercises at home at least six times per week. When a patient was unable to do so, she was excluded from the study.

A paper booklet and a YouTube video were provided to help them perform exercises accordingly. When needed, telephone and walk-in advice were offered.

Informed consent statement: An informed consent form was prepared, which had to be signed by all the subjects participating in the study who previously received sufficient information about the objectives and the procedure of the study. They were also informed of the possibility of revoking the consent given at any time without having to justify their decision without prejudice. All necessary permits were requested from the institutions for the development of the research.

## Results

Recruitment: Patients attending the Lymphoedema Unit with clinical manifestations of AWS (whose functionality, quality of life, and exercise capacity are widely depleted). Enrollment began in October 2021 until September 2024. Forty-six patients participated, 24 in the intervention group and 22 in the control group (Fig. 1).

The descriptive analysis of the outcomes included in the study is presented in Table 1, showing both quantitative and qualitative outcomes. Quantitative data are expressed as mean ± standard deviation, median [Q1–Q3], and minimum–maximum values, while qualitative data are presented as percentages along with their 95% confidence intervals.

The bio-demographic data of all participants at baseline showed homogeneity and non-significant differences between the two groups in terms of age, weight, height, body mass index, type of oncological treatment, type of surgical treatment, stage, and type of cancer. No type 1 error could be detected (Table 1).

For quantitative outcomes, Wilcoxon tests were conducted. *p-value*, probability value; *N*, population size; ±, range of values or a confidence interval; *TNM*, TNM staging system is a method for classifying cancer based on the extent of the tumor, the presence of regional lymph node involvement, and the presence of distant metastasis; *X*, mean; *SD*, standard deviation.

**Fig. 2:**
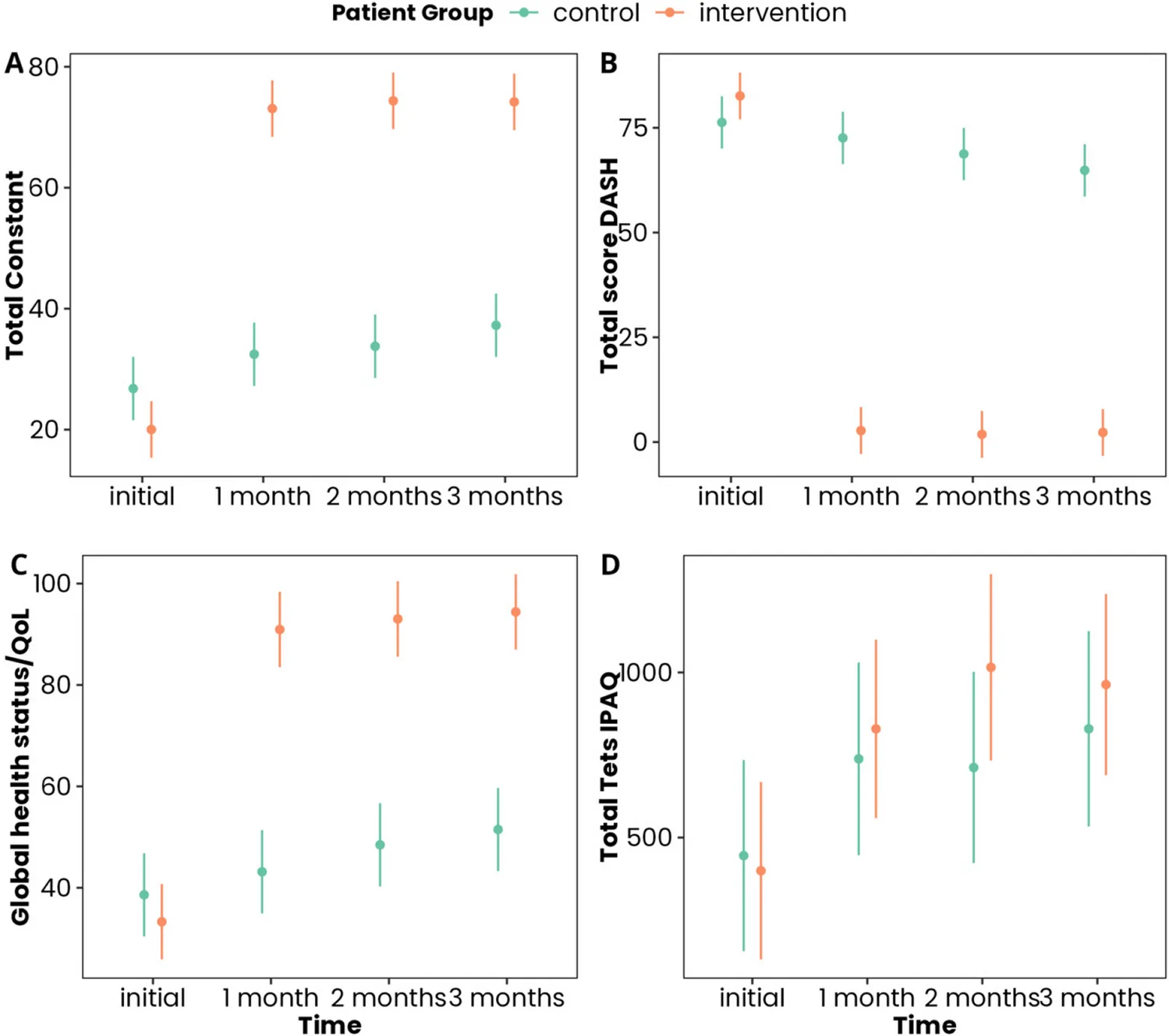
Changes in the total score of **A** Constant-Murley test, **B** DASH test, **C** EORTC QLQ-BR23, and **D** IPAQ as a function of intervention group and time of measurement. Mean and standard error are represented

There is a significant association between the total DASH test score and the time of test measurement according to the intervention group. On one hand, as observed in Fig. 2B, patients in the intervention group have a significant decrease in the test score from the first month, maintaining this improvement in the rest of the visits (*p* < 0.001). On the other hand, patients in the control group show a more gradual decrease in test score, never reaching the scores of the intervention group (Table 2). The same occurs using the Constant-Murley test (*p* < 0.001) (Fig. 2A, Table 3) as well as the EORTC-QLQ-BR23 questionnaire (*p* < 0.001) (Fig. 2C, Table 4).

**Table 2:** Estimated marginal means (EMM) and Tukey’s post hoc test of the Disabilities of the Arm, Shoulder and Hand (DASH) score as a function of intervention group and control group, and time of assessment. Significant results are indicated with asterisks: *p* < 0.001: ***; *p* < 0.01: *; *p* < 0.05:; *p* < 0.1:

**EMM**				
	**Control**		**EMM ± SE**	**CI 95%**
		Initial	76.29 ± 3.11	[70.06–82.53]
		1 month	72.58 ± 3.11	[66.34–78.82]
		2 months	68.75 ± 3.11	[62.52–74.99]
		3 months	64.85 ± 3.11	[58.62–71.09]
	Intervention			
		Initial	82.61 ± 2.79	[77.02–88.2]
		1 month	2.75 ± 2.79	[− 2.84–8.34]
		2 months	1.84 ± 2.79	[− 3.75–7.44]
		3 months	2.3 ± 2.79	[− 3.29–7.89]
Comparisons				
	Control		**Difference ± SE**	***p*-value**
		Initial – 1 month	3.71 ± 2.21	0.338
		Initial – 2 months	7.54 ± 2.21	0.005 **
		Initial – 3 months	11.44 ± 2.21	< 0.001 ***
		1 month – 2 months	3.83 ± 2.21	0.312
		1 month – 3 months	7.73 ± 2.21	0.004 **
		2 months – 3 months	3.9 ± 2.21	0.295
	Intervention			
		Initial – 1 month	79.86 ± 2.12	< 0.001 ***
		Initial – 2 months	80.76 ± 2.12	< 0.001 ***
		Initial – 3 months	80.31 ± 2.12	< 0.001 ***
		1 month – 2 months	0.9 ± 2.12	0.974
		1 month – 3 months	0.45 ± 2.12	0.997
		2 months – 3 months	− 0.45 ± 2.12	0.997
	Between group comparison (control—intervention)			
		Initial	− 6.32 ± 3.71	0.094
		1 month	69.83 ± 3.71	< 0.001 ***
		2 months	66.91 ± 3.71	< 0.001 ***
		3 months	62.56 ± 3.71	< 0.001 ***

*P*-value Probability value,* N* Population size, *±* Range of values or a confidence nterval, *EMM* Estimated Marginal Mean *SE* Standard Error, *CI *Confidence Interval. * Asterisk indicates statistical significance

**Table 3:** 0.1: ·Estimated marginal means (EMM) and Tukey’s post hoc test of the Constant-Murley Score by intervention group and control group (control vs. intervention) and measurement timepoint. Significant results are indicated with asterisks: p < 0.001: ***; p < 0.01: **; p < 0.05: *; p <

**EMM**				
	**Control**		**EMM ± SE**	**CI 95%**
		Initial	26.79 ± 2.6	[21.55–32.03]
		1 month	32.47 ± 2.6	[27.23–37.7]
		2 months	33.78 ± 2.6	[28.55–39.02]
		3 months	37.25 ± 2.6	[32.01–42.49]
	Intervention			
		Initial	20.02 ± 2.33	[15.34–24.7]
		1 month	73.08 ± 2.33	[68.4–77.76]
		2 months	74.37 ± 2.33	[69.69–79.05]
		3 months	74.19 ± 2.33	[69.51–78.87]
Comparisons				
	Control		Difference ± SE	***p***-value
		Initial – 1 month	− 5.68 ± 1.5	0.001 **
		Initial – 2 months	− 6.99 ± 1.5	< 0.001 ***
		Initial – 3 months	− 10.46 ± 1.5	< 0.001 ***
		1 month – 2 months	− 1.32 ± 1.5	0.816
		1 month – 3 months	− 4.78 ± 1.5	0.01 **
		2 months – 3 months	− 3.46 ± 1.5	0.101
	**Intervention**			
		Initial – 1 month	− 53.06 ± 1.46	< 0.001 ***
		Initial – 2 months	− 54.35 ± 1.46	< 0.001 ***
		Initial – 3 months	− 54.17 ± 1.46	< 0.001 ***
		1 month – 2 months	− 1.29 ± 1.46	0.812
		1 month – 3 months	− 1.11 ± 1.46	0.873
		2 months – 3 months	0.19 ± 1.46	0.999
	**Between group comparison (control—intervention)**			
		Initial	6.77 ± 3.04	0.031*
		1 month	− 40.62 ± 3.04	< 0.001 ***
		2 months	− 40.59 ± 3.04	< 0.001 ***
		3 months	− 36.94 ± 3.04	< 0.001 ***

*P *value Probability value,* N* Population size , ± Range of values or a confidence nterval, *EMM* Estimated Marginal Mean, *SE *Standard Error, *CI* Confidence Interval, *: Asterisk indicates statistical significance.

**Table 4:** Estimated marginal means (EMM) and Tukey’s post hoc test of EORTC QLQ-BR23 test score by intervention group and control group (control vs. intervention), and measurement timepoint. Significant results are indicated with asterisks: *p* < 0.001: ***; *p* < 0.01: **; *p* < 0.05: *; *p* < 0.1: ·

**EMM**				
	**Control**		**EMM ± SE**	**CI 95%**
		Initial	38.61 ± 4.11	[30.41–46.82]
		1 month	43.16 ± 4.11	[34.95–51.36]
		2 months	48.46 ± 4.11	[40.26–56.67]
		3 months	51.49 ± 4.11	[43.29–59.7]
	**Intervention**			
		Initial	33.3 ± 3.73	[25.87–40.73]
		1 month	90.94 ± 3.73	[83.51–98.37]
		2 months	93.02 ± 3.73	[85.59–100.45]
		3 months	94.41 ± 3.73	[86.98–101.84]
**Comparisons**				
	**Control**		**Difference ± SE**	***p*-value**
		Initial – 1 month	− 4.55 ± 3.74	0.618
		Initial – 2 months	− 9.85 ± 3.74	0.046*
		Initial – 3 months	− 12.88 ± 3.74	0.004**
		1 month – 2 months	− 5.3 ± 3.74	0.491
		1 month – 3 months	− 8.33 ± 3.74	0.121
		2 months – 3 months	− 3.03 ± 3.74	0.85
	**Intervention**			
		Initial – 1 month	− 57.64 ± 3.58	< 0.001 ***
		Initial – 2 months	− 59.72 ± 3.58	< 0.001 ***
		Initial – 3 months	− 61.11 ± 3.58	< 0.001 ***
		1 month – 2 months	− 2.08 ± 3.58	0.937
		1 month – 3 months	− 3.47 ± 3.58	0.767
		2 months – 3 months	− 1.39 ± 3.58	0.98
	**Between group comparison (control—intervention)**			
		Initial	5.31 ± 5.03	0.294
		1 month	− 47.78 ± 5.03	< 0.001 ***
		2 months	− 44.56 ± 5.03	< 0.001 ***
		3 months	− 42.92 ± 5.03	< 0.001 ***

*P* value Probability value, *N *Population size, ± Range of values or a confidence interval, *EMM* Estimated Marginal Mean, *SE* Standard Error,* CI* Confidence Interval, * Asterisk indicates statistical significance

There is a significant association between the total IPAQ test score and the time of test measurement. All patients, both intervention and control groups, significantly increase their physical activity from the first month onwards, maintaining these activity levels over time (Fig. 2D).

Complications during the treatment: Two of the patients in the intervention group developed a slight thickening around the forearm over the cord. This disappeared spontaneously after a few days.

## Discussion

The main finding of this study was that 22 out of the 24 patients in the intervention group did not suffer from AWS at the end of the treatment. The remaining 2 patients, although still suffering from AWS, regained functionality, as well as a substantial improvement in their quality of life. On the contrary, all patients in the control group still suffered AWS on the 90th day follow-up, showing limited mobility, pain, impaired function, and impaired quality of life. Of the 46 participants included, only one suffered grade 2 lymphoedema, and in this case, she belonged to the intervention group. Her lymphoedema did not prevent her from performing the programmed activity within such group.

Almost all randomized clinical trials that included the functionality outcome conclude that physical therapy allows the finding of statistically significant differences for the intervention group (*p* < 0.05) [6–8, 18, 19]. The DASH scale is the most used [6, 16, 18, 19]. The type of physical therapy applied was manual lymphatic drainage combined with physical therapy (stretching, strengthening, and active exercises) [6, 7, 18], early physical therapy combined with educational tips [8]. Klein et al. [19] added scar massage to the aforementioned therapies. One study used myofascial release combined with moist heat [16]. Some authors found significant differences in the short term (at the primary and at the 3-month follow-up), but no significant differences were found at the 6-month follow-up [7]. However, other authors describe that in small (*p* = 0.004) and extensive surgeries (*p* = 0.032), additional positive effect for the intervention 6 months postoperatively on functional disability was revealed [19].

The vast majority of studies that consider quality of life outcome used the EORTC QLQ-C30/BR23 scale (Meer, Yuste, Cho, Muñoz) [6, 8, 18, 30]. Only the study by Meer et al. [6] found significant differences in the Global Score of the scale. Yuste et al. [18, 30] and Cho et al. [18] found significant differences in the physical and social dimensions of the scale, and Muñoz et al. [8], Cho et al. [18], and Torres et al. [7] found those significant differences in the function dimensions of the scales used. Some authors found significant differences in the short term (at the primary and at the 3-month follow-up), but no significant differences were found at the 6-month follow-up [7].

Regarding the outcome exercise capacity, at present, no study that includes this outcome for patients with AWS after breast cancer has been found. There are some studies that use the dynamometer for the strength outcome, but none use a validated scale for exercise capacity (Muñoz, Ibrahim, Cho) [8, 18, 20]. Our study is a pioneer in this respect.

The correct performance of the exercises together with adherence is considered key factors in the reliability on these study results. Torres et al. [7] through their study also showed good and parallel adherence in both groups. Klein et al. [19] offered a paper booklet and phone call assessment 1 week and 1 month after the intervention. Cho et al. [18] focused on the support for the physical therapy and manual lymphatic drainage group during the first week. This posed a confusing factor in the perception of pain within such group. Their study neither detailed the applied exercises nor the intervention group patients’ adherence. Moreover, the study by Cho et al. [18] considered unethical the presence of a control group, disregarding it for the study. As a result, this study showed a big dropout rate, with a total loss of 29,out of the initial 70 patients within the intervention group.

There are other studies with a high drop-out percentage. This may pose a challenge in the reliability of the results. Meer et al. [6] started with 36 patients enrolled, and only 20 finished after the whole period. At the end of the 18 month follow-up, the study by Ibrahim et al. [17] could only count on 33 out of the 59 patients who initially started the clinical trial. Starting with 31 patients, the study of Muñoz et al. [8] could only count on 12 patients at the end of it. Nevertheless, there are other researches with follow-up periods for over a year without such a high drop-out rate [20, 30].

Publications showed the need for a treatment protocol of choice for AWS as there is currently none. AWS experts emphasize the need for more randomized clinical trials, as the current clinical trials provide a high heterogeneity of physiotherapy treatments [5, 11, 21, 31]. Also, some contain a very small sample size [6, 16].

### Strengths and limitations

- Not only adherence to the scheduled activity was very good in both groups with almost no loss of recruited patients but also the physiotherapist made sure that the scheduled exercises were performed correctly.
- Considering that all outcomes showed good results for the intervention group from the first follow-up on day 30 after the first examination, this physiotherapy technique could be considered treatment of choice for AWS.
- In order to reinforce its external validity, it is highly recommended to perform this study as a multi center research.
- Due to the nature of the intervention, blinding is challenging. Normally, in physiotherapy research, it is difficult to mask the group to which patients belong.
- Future directions: 

Ideally, future studies should include imaging tests, such as indocyanine green lymphography [32] or lymphoscintigraphic imaging [15], in order to better understand the process of making AWS disappear. Only a few studies use imaging techniques that visualize AWS, and of those, some seem to describe that the cord disappears and new recanalizations and collateralizations appear [15].

## Conclusions

The results suggest that stretching combined with scar massage and manipulative tissue release techniques leads to a better recovery and improves functionality and quality of life in patients suffering from axillary web syndrome. The physiotherapy approach described in this article could be the method of choice for this surgical sequela.

## Supplementary Information

Below is the link to the electronic supplementary material.

ESM 1 (DOC 219 KB)

## Data Availability

No datasets were generated or analysed during the current study.

## Declarations

**Ethical approval.**

Ethics approval was granted by the Andalusia Ethics Committee (PEIBA code 1909-N1-21, reg. number 171.21).

## Protocol

The study protocol was approved by the Andalusia Ethics Committee.
